# Artificial Feeding of All Consecutive Life Stages of *Ixodes ricinus*

**DOI:** 10.3390/vaccines9040385

**Published:** 2021-04-14

**Authors:** Nina Militzer, Alexander Bartel, Peter-Henning Clausen, Peggy Hoffmann-Köhler, Ard M. Nijhof

**Affiliations:** 1 Institute of Parasitology and Tropical Veterinary Medicine, Freie Universität Berlin, 14163 Berlin, Germany; Nina.Militzer@fu-berlin.de (N.M.); Peter-Henning.Clausen@fu-berlin.de (P.-H.C.); peggy.koehler@fu-berlin.de (P.H.-K.); 2 Institute for Veterinary Epidemiology and Biostatistics, Freie Universität Berlin, 14163 Berlin, Germany; alexander.bartel@fu-berlin.de

**Keywords:** *Ixodes ricinus*, artificial tick feeding, in vitro tick feeding, vitamin B, life cycle

## Abstract

The hard tick *Ixodes ricinus* is an obligate hematophagous arthropod and the main vector for several zoonotic diseases. The life cycle of this three-host tick species was completed for the first time in vitro by feeding all consecutive life stages using an artificial tick feeding system (ATFS) on heparinized bovine blood supplemented with glucose, adenosine triphosphate, and gentamicin. Relevant physiological parameters were compared to ticks fed on cattle (in vivo). All in vitro feedings lasted significantly longer and the mean engorgement weight of F_0_ adults and F_1_ larvae and nymphs was significantly lower compared to ticks fed in vivo. The proportions of engorged ticks were significantly lower for in vitro fed adults and nymphs as well, but higher for in vitro fed larvae. F_1_-females fed on blood supplemented with vitamin B had a higher detachment proportion and engorgement weight compared to F_1_-females fed on blood without vitamin B, suggesting that vitamin B supplementation is essential in the artificial feeding of *I. ricinus* ticks previously exposed to gentamicin.

## 1. Introduction

Ticks are obligate hematophagous arthropods and divided in three families: hard ticks (Ixodidae), soft ticks (Argasidae), and the monotypic Nuttalliellidae [1,2,3]. About ten percent of the approximately 900 known tick species are of medical or veterinary relevance and may cause direct damage due to their blood feeding habit, as well as indirect damage by acting as vectors for pathogens, including viruses, bacteria, and protozoan parasites [4,5]. In the northern hemisphere, four tick species belonging to the *Ixodes ricinus* species complex: *I. ricinus*, *I. scapularis*, *I. pacificus,* and *I. persulcatus* are of particular relevance as they may act as vectors for a number of zoonotic pathogens, including *Borrelia burgdorferi* sensu lato. In Europe, *I. ricinus* is widely distributed and can serve as a vector for other human pathogens, such as tick-borne encephalitis virus, *Babesia divergens,* and *Anaplasma phagocytophilum* as well [6,7]. *Ixodes ricinus* is a three-host tick species; all life stages (larvae, nymphs and adult females) require a blood meal from different hosts for their development. This tick species also has an extraordinary broad host range on which it can feed, ranging from small mammals to livestock, birds, reptiles and humans [8,9].

To facilitate research on hematophagous arthropods, such as mosquitoes, flies, and ticks, in vitro feeding techniques have found wide application [10,11,12,13,14]. In addition, they also contribute to the 3R principle to reduce, replace, and refine the use of animals in research. Artificial tick feeding systems (ATFS) have also found increased use in recent years to study tick biology, tick-pathogen interactions, drug development, and development of anti-tick vaccines under defined laboratory conditions [15,16,17,18,19,20]. ATFS also found a wide application for identifying different tick control targets or the development of anti-tick vaccines under defined laboratory conditions [17,18,19,20].

Feeding systems for hematophagous arthropods typically consists of four parts: (1) a unit containing the arthropods, (2) the blood meal, (3) a membrane that mimics skin and separates the arthropods from the blood meal, and 4) a temperature control system to heat the blood meal to a temperature corresponding to the body temperature of their homeothermic hosts [21,22,23]. 

In contrast to soft ticks and other hematophagous arthropods, which generally feed for short periods only, hard ticks feed for prolonged periods of up to several days or weeks; *I. ricinus* juvenile ticks typically feed for 3–5 days and adults for 7–12 days [24,25]. This long duration forms a major challenge in the artificial feeding process [26], as it, in combination with temperatures of approximately 37 °C, results in a higher risk of decay of the blood meal. This results in the need for regular blood changes, making the artificial feeding a laborious process, and the addition of antibiotics in the blood meal, which may affect the tick microbiome including nutritive symbionts [12,27,28,29,30]. In addition, hard ticks also have an intricate pre-feeding behavior [24,26], the mimicking of which in vitro can be complicated. 

Hard ticks including *I. ricinus* are commonly reared on experimental animals [31], although reports on the in vitro feeding of nymph and adult *I. ricinus* have been published [15,32,33,34]. The only hard tick species for which successful feeding of all life stages has previously been reported is the tropical bont tick *Amblyomma hebraeum* [27]. Here, we report on the completion of the life cycle of *I. ricinus* in vitro by the feeding of all consecutive life stages using an ATFS and a comparison between relevant biological parameters of in vitro fed ticks and ticks fed on cattle, further referred to as in vivo fed ticks. 

## 2. Materials and Methods

### 2.1. Tick Feeding

All ticks used for this study originated from a laboratory colony at the Institute of Parasitology and Tropical Veterinary Medicine of the Freie Universität Berlin. For the maintenance of this colony, larvae are routinely fed on laboratory gerbils (*Meriones unguiculatus*), nymphs on rabbits (*Oryctolagus cuniculus*), and adults on rabbits or calves (*Bos taurus*). All replete larvae and nymphs are kept at room temperature and >90% Relative Humidity (RH). Shortly after molting into the adult stage, ticks are separated by sex and stored at 12 °C and >90% RH in the dark. Replete adult ticks are kept in the dark at 20 °C, >90% RH. 

For this study, 8 to 32-week-old and 8 to 15-week-old larvae were used for the in vivo and in vitro feeding, respectively. In vivo fed nymphs were fed at an age of approximately 2–4 months post molt, in vitro nymphs at 2–3 months. In vitro F_0_-adult ticks were fed at an age of 7–9 months post molt, while in vitro F_1_-adult ticks were fed at 5–7 months. Adults fed in vivo had an age range of 2–10 months post molt.

For our study, all life stages reported as in vivo fed ticks were fed inside linen ear bags on 14 to 18-week-old tick-naïve Holstein-Friesian calves. The ears were checked twice daily for engorgement starting at three or five days post-infestation for juvenile and adult ticks, respectively. Thirty female and 30 male *I. ricinus* adults, nine months after being fed on rabbits, were used to initiate the in vitro life cycle (F_0_ adults). They were brought together in a desiccator kept at room temperature and >90% RH seven days before the start of the artificial feeding.

Blood used for in vitro feeding was drawn aseptically from cattle that grazed on pastures considered to be free of ticks; natural tick infestations were not observed during this period. All animal experiments were approved by the regional authorities for animal experiments (Landesamt für Gesundheit und Soziales, Berlin, Germany, 0387/17). 

### 2.2. Artificial Tick Feeding System (ATFS)

The ATFS developed by Kröber and Guerin [1] was adapted as previously reported [35]. For containment of the blood, autoclaved 50 mL beaker glasses (SIMAXX, Bohemia Cristal, Selb, Germany) or sterile standard 6-well cell culture plates were used. Ten females and 10 males were placed in each feeding unit. Juvenile ticks were fed using a smaller feeding system, which fitted in a standard 12-well cell culture plate (Sarstedt, Nürnbrecht, Germany). Here, the feeding units were made up of a borosilicate glass tube (length 40 mm, inner diameter 15–16 mm; Neubert Glass Geschwenda, Germany and Glastechnik Rahm Mutterz GmbH, Switzerland), and a smaller rubber ring with an inner diameter of 18 mm (Emil Lux GmbH, Wermelskirchen, Germany). A moistened air-permeable foam plug (K-TK e.K., Retzstadt, Germany) was used instead of a plastic stopper for the smaller feeding units. 

### 2.3. Artificial Membranes

The silicone mixture for the artificial membranes was produced as previously described [32,35]. A metal scraper (Emil Lux) was used to spread the silicone paste onto a matrix made of lens cleaning paper for adult ticks (Tiffen, Happauge, NY, USA) or goldbeater’s membrane for juvenile ticks (20 µm thickness, Altenburger Pergament and Trommelfell GmbH, Altenburg, Germany). After overnight drying, the membranes were glued to glass tubes using silicone glue (Elastosil E41, Wacker, München, Germany). The membrane thickness varied from 40–50 μm, 50–70 μm, and 80–120 μm for larvae, nymphs, and adults, respectively. In the adult ATFS, a piece of 15 × 20 mm of glass fiber netting (Drahtwaren Driller GmbH, Freiburg, Germany) was glued on top of the membrane to provide a mechanical attachment stimulus. The feeding units were tested for leakage and disinfected for >10 min using ethanol (70%), followed by autoclaving of adult feeding units before use. 

### 2.4. Animal Hair Extract

Membranes were treated with 0.35 mg (juvenile ATFS) or 0.525 mg (adult ATFS) of a low volatile mass (LVM) animal hair extract to increase their attractivity for ticks. This extract was prepared by immersing 50 g of freshly collected animal hair in a 2:1 chloroform-methanol mixture for 2 h, followed by immersion in a 1:1 chloroform-methanol mixture for a 2 h and a third immersion in a 1:2 chloroform-methanol mixture at 45 °C. The supernatant was collected after each immersion, vacuum filtered and subsequently concentrated by roto-evaporation. The extract was finally dissolved in a 1:2 chloroform-methanol mixture and stored at −80 °C. Extracts were diluted to appropriate working concentrations and stored at −20 °C. After application on the membrane, the extract was allowed to evaporate for at least 2 h before ticks were placed in the feeding unit. 

### 2.5. Blood Meal and the Artificial Tick Feeding Procedure

Collected bovine blood was immediately treated with 20 IU/mL sodium-heparin (B. Braun, Melsung, Germany) and 2 g/L sterile glucose and stored at 4 °C for a maximum of one week. Prior to each blood change, 0.1M adenosine triphosphate (ATP, Carl Roth, Karlsruhe, Germany) dissolved in 0.9% autoclaved NaCl (VWR, Darmstadt, Germany), and 5 μg/mL gentamicin (Cellupur, Roth) were added to the blood, which was subsequently warmed to 37 °C in a water bath. The culture plates were warmed to ~37 °C using a heating plate (Hot Plate 062, Labotect, Göttingen, Germany). During the blood change, which took place every 10–14 h, the underside of the membrane was cleaned using sterile 0.9% NaCl and the feeding unit was transferred to a new cell culture plate containing freshly prepared blood meals. In general, in vitro tick feeding was performed in an incubator (ICH110C, Memmert, Schwabach, Germany) set at 20 °C, 80% RH and 4% CO_2_. Adults were fed under a 16:8 light-dark-cycle, whereas juvenile stages were fed in the dark. For the in vitro F_1_-adult feeding, ticks were split in different groups, which were either fed in autoclaved sterile 50-mL beaker glasses in a water bath (WNE 7, Memmert) heated to 37 °C or in an incubator as described above. Furthermore, for one group of F_1_-adults the blood meal was supplemented with an aqueous sterile filtered solution of ten vitamin B components [29]. For each in vitro experiment, the temperature and RH inside the feeding units were monitored by an iButton data logger (Maxim Integrated, San Jose, CA, USA) placed in a control feeding unit without ticks.

### 2.6. Data Collection of In Vitro and In Vivo Feedings

All in vitro feeding experiments were initiated in the evening. The tick feeding units were inspected with each blood change and the number of attached ticks was documented twice daily. Observations on the larvae started three days after the larvae were placed in the feeding units. For in vivo feeding experiments, the approximate number of larvae placed on an animal was calculated by dividing the weight of the larval batch with the calculated mean weight of a single unfed larva, as previously measured in batches on an analytic scale (Ohaus Discovery, Nänikon, Switzerland). After detachment, the juveniles were washed in water and air-dried. The detachment weight was measured using a balance scale (LC220S, Sartorius GmbH, Göttingen, Germany) for adults and an analytic scale for larvae and nymphs, whereby larvae were weighed in batches. All detachment weights were measured within 24 h. Only females with a weight higher than the female with the lowest detachment weight that still produced viable larvae, were taken into consideration for further analyses. 

For individual females, the pre-oviposition period as well as the time between engorgement and hatching of the first larvae (pre-hatch period) were recorded. The egg batch mass was measured by an analytic scale after the first larvae had hatched or 80 days after female detachment. The hatching was scored qualitatively by estimation under stereo microscope (1: ≈0%, 2: ≈5%, 3: ≈50%, 4: ≈75%, 5: ≈100% hatching). The egg conversion ratio was calculated as egg mass divided by female engorgement weight. Molted adults were separated by sex and weighed using an analytic scale.

### 2.7. Video 

Exemplary video recording of nymphs inside feeding units were done using a Wi-Fi otoscope camera (SB-10, Bysameyee, Shenzhen Shengyi Electronic Commerce Co, Ltd., Guangdongsheng, China) and edited in iMovie (10.1.6, Apple Inc., Cupertino, CA, USA). 

### 2.8. Statistics 

Statistical analyses and graphs were made in R version 3.6.0. For graphs, the ggplot2-package (3.3.1) [36] and the 95% confidence intervals of proportions were computed by binom.wilson from the “epitools”-package (version 0.5–1.0). Depending on normal distribution, statistical differences were calculated by either *t*-test with Welch correction or Mann–Whitney U test. For proportions of attachment, detachment, engorgement, molting, oviposition, female molting, and of deployed ticks reaching the next life stage, the Z-test was performed. Results are reported with 95% confidence intervals (CI), standard deviation or coefficient of variation (CV). For larvae hatch steps, median and interquartile range (IQR) were computed. A significance threshold of 0.05 was used. For adult ticks, individuals were taken into considerations, while for juveniles, depending on the feeding parameters either individual ticks, feeding unit batches or whole experimental replicates were taking into consideration.

To further quantify the effect of in vitro feedings on detachment weight for adults, egg batch mass and proportion of viable larvae-producing females (number of initially fed females/number of viable larvae batches), different mixed models by the glmer.nb function for count data due to overdispersion or lmer function for continuous data from the “lme4”-package (version 1.1–26) with a nested design were constructed. As fixed effect, the in vivo or in vitro feeding was assessed. To study the F_1_-in vitro feeding, further fixed effects were considered: generation (F_0_/F_1_), vitamin B-supplementation and incubator/water bath feeding method. To account for repeated measures nested random effects for experiment treatment (in vitro/in vivo), experimental replicates (in vivo: 5, in vitro F_0_: 1, in vitro F_1_: 2), experimental units (in vivo: 1–2, in vitro F_0_: 3, in vitro F_1_: 1–2) were included. 

## 3. Results

### 3.1. Feeding of F_0_ Adult Ticks

The batch of F_0_ adult ticks used to initiate the in vitro life cycle were fed in vitro in the winter season and had a maximum attachment of 20%. F_0_-females only attached and engorged in two out of three feeding units, attachment was not observed in the third feeding unit. For comparison purposes, 450 female and 450 male ticks were fed in vivo on calves, in batches of 50 females and 50 males per ear between August 2018 and August 2020. An overview of the adult in vitro and in vivo feeding is shown in Table 1.

**Figure 1:**
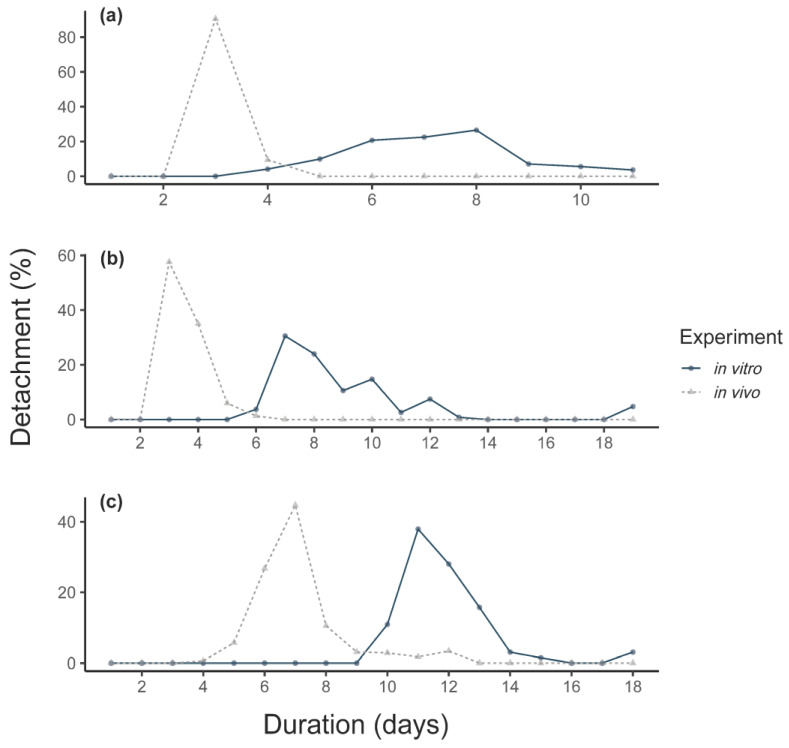
Mean feeding duration of (**a**) larvae, (**b**) nymphs and (**c**) adults (F_0_ + F_1_) in vitro compared to in vivo fed ticks fed on calves (means). Detachment is presented as a % of all detached ticks.

In general, in vivo F_0_ adult females reached a higher detachment weight (231 ± 72.3 vs. 136 ± 44.9 mg) and egg mass (116 ± 39.5 vs. 46 ± 27.8 mg) than in vitro F_0_ females. The mean detachment weight in vivo was thereby 94 mg higher (linear mixed-effect model (LMM), CI: 21.33–166.66, *p* = 0.011) and the egg mass weight oviposited by the in vivo fed females was 69 mg higher (LMM, CI: 21.69–116.81, *p* = 0.004, number of observations (obs): 256). All in vitro feeding durations were longer compared to in vivo feeding (Figure 1). The duration of the in vitro feeding was significantly longer than the in vivo feeding (Mann–Whitney U-test, *p* < 0.0001, Figure 1c). However, the pre-oviposition period was significantly shorter for in vitro fed ticks, as was the mean pre-hatch period (Table 1).

### 3.2. Feeding of Larvae

F_1_ larvae in vitro feeding was performed in two experimental replicates with six feeding units each during the summer season. A total of 1003 larvae were fed in vitro with an average of 84 (CI: 64–103) larvae per feeding unit. To calculate the approximate number of in vivo fed larvae, the mean weight of unfed larvae was measured in batches of 83–103 larvae. Here, the mean weight of a single unfed larva was calculated to be approximately 0.0223 ± 0.0012 mg. Based on this calculation, approximately 11,737 larvae with a mean of 2347 (CI: 1044–3651) larvae were fed per calf’s ear in three independent experiments in spring, summer, and autumn season. 

**Figure 2:**
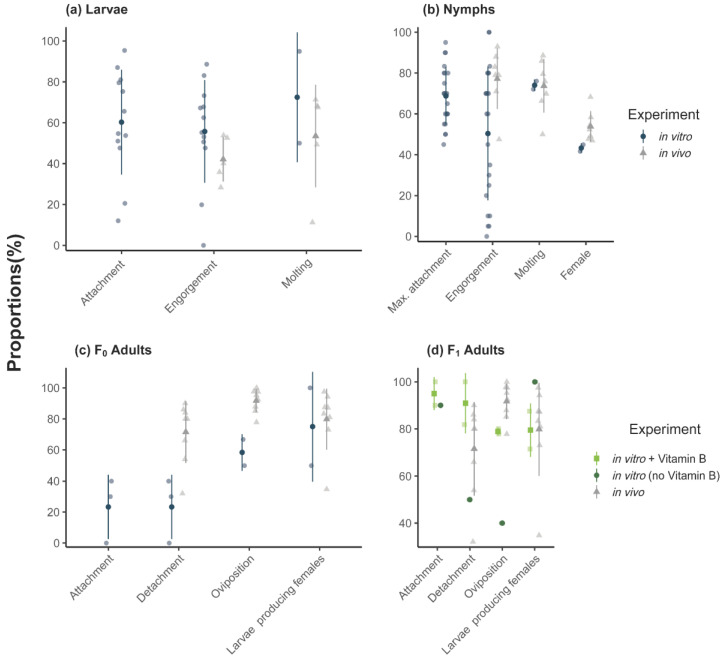
Mean proportions (%) per feeding unit for (**a**) larvae, (**b**) nymphs and (**c**) F_0_ adults and (**d**) F_1_ adults (with and without vitamin B) in vitro compared to in vivo (means ± SD). Molting proportion: molted ticks per engorged tick; oviposition proportion: egg batches per detached female; larvae production: viable larvae producing females per egg batch.

For the in vitro ticks, an average attachment proportion of 60% was observed, with a range of 12–95% per feeding unit (Figure 2). In vitro fed larvae showed a significant higher engorgement and molting proportion compared to in vivo fed larvae (Table 2). However, the feeding duration of larvae fed in vitro was longer compared to in vivo fed larvae (Figure 1a). For larvae, contamination occurred for 3/12 feeding units after ~7 days of feeding. A total of 446 nymphs (44%) successfully molted from the in vitro fed larvae.

### 3.3. Feeding of Nymphs

A total of 426 nymphs were fed in vitro at two occasions with a total of 21 feeding units containing 20 nymphs each and one unit containing six nymphs. The in vitro feeding of nymphs was performed in autumn. For the in vivo feeding of nymphs, a total of 800 nymphs were fed on four occasions with 100 nymphs per calf’s ear in spring, summer, and autumn.

For the in vitro feeding, maximum nymphal attachment was observed after an average of four days. The first engorged nymphs per unit were collected after a mean of 6.9 days, significantly later than in vivo (2.9 days, Figure 1b). Leakage occurred in 3/21 feeding units and visible contamination was observed in 57% (12/21) of the feeding units.

The proportion of nymphs that engorged in vitro was lower than in vivo (Figure 2b, Table 3) as was their engorgement weight (in vitro: 2.82 mg, in vivo: 3.32 mg). The weights of both males (in vitro: 0.81 mg, in vivo: 0.98 mg) and females (in vitro: 1.32 mg, in vivo: 1.68 mg) that molted from in vitro engorged nymphs was significantly lower compared to the weights of adults fed as nymphs in vivo as well. A total of 157 (67 females and 90 males) F_1_-adults successfully molted from the in vitro fed nymphs. 

### 3.4. Feeding of F_1_-Adults In Vitro

The 51 F_1_-females were split in groups. The first group of 20 females was fed under the same conditions as the F_0_-adults. Although the proportion of attached females was 75% (CI: 41–100), only one female detached after 13 days with a detachment weight of 55 mg. She produced a viable egg batch of 14 mg that started hatching after 68 days. Almost all eggs hatched successfully. 

The second and third F_1_-adult group were given a blood meal supplemented with vitamin B (*n* = 21 females) or without vitamin B (*n* = 10 females). Both groups were fed in a water bath system (Table 1). 

**Figure 3:**
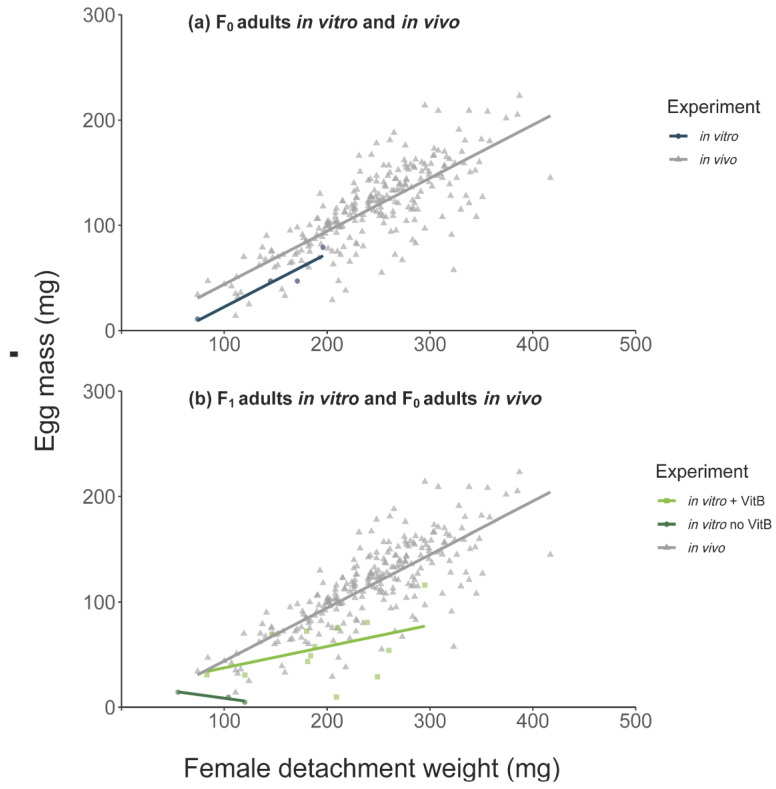
Egg mass (mg) and female detachment weight (mg) from (**a**) F_0_ in vitro and in vivo ticks and (**b**) F_1_ in vitro (water bath, with and without vitamin B) and F_0_ in vivo-fed ticks.

The mean detachment weight and mean egg mass were higher for in vivo fed females (Figure 3). The effect of vitamin B supplementation on female detachment weight and egg mass are presented in Table 4 (with F_1_-adults with vitamin B in a water bath environment as intercept). There were significantly lower detachment weights for F_1_ adults fed in vitro without vitamin B regardless of incubator or water bath feeding (Table 4). We also observed a negative effect of the absence of vitamin B supplementation on egg masses and on the proportion of females producing viable larvae batches. Only 9% of viable larvae batches were produced by F_1_-females fed without vitamin B in the incubator (generalized linear mixed-effect model (GLM), 0.09, CI: 0.01–0.64 *p* = 0.017) and only 35% of viable larvae batches were produced by F_1_-females fed without vitamin B in a water bath environment (GLM, 0.35, CI: 0.08–1.56, *p* = 0.168).

The addition of vitamin B to the blood meal in ticks fed in a water bath environment resulted in increased detachment and egg mass, although these were still lower than those observed for in vivo fed ticks (Table 4). The positive effect of vitamin B supplementation in a water bath environment to F_1_ ticks was also observed for the proportion of females producing viable larvae batches, which were not statistically significant from the in vivo group (GLM, 0.89, CI: 0.41–1.91, *p* = 0.763).

It was observed on several occasions that attached females started to turn black after ~7 days of feeding and soon thereafter died. This occurred for 13/15 attached females fed on blood without vitamin B supplementation in an incubator and for 2/9 females fed on blood without vitamin B supplementation in a water bath, but was not observed for the 20 females that successfully engorged on a blood meal with vitamin B supplementation in a water bath environment. 

## 4. Discussion

Over 25 years ago, the completion of the life cycle of *Amblyomma hebraeum* in vitro was reported [27], which until now remained the only ixodid tick species for which this was done. Although *Ixodes* nymphs and adults have successfully been fed in vitro using silicone-based membranes [19,28,32,33,37,38,39,40], only prepared animal skin membranes have previously been used for the artificial feeding of *I. ricinus* larvae [41]. We here report on the feeding of all consecutive *I. ricinus* life stages using a modified ATFS and include a comparison to *I. ricinus* ticks fed on calves. 

### 4.1. Feeding Duration

Generally, hard ticks feed for longer periods than soft ticks [26]. In our study, in vivo fed ticks exhibited similar feeding durations as previously reported [24,25,42,43]. Significantly longer feeding durations were observed for all *I. ricinus* life stages fed in vitro. This finding was most apparent in F_0_ adults and correspond to previous studies on *Ixodes* spp. [28,38,40]. Reports on feeding duration of in vitro fed ticks are quite heterogeneous, possibly due to differences in the ATFS, blood meal diets, season, tick fitness, as well as differences between tick species and their feeding behavior. It appears, for example, that adults of several metastriate tick species do not exhibit prolonged feeding durations when compared to in vivo feeding [27,44,45]. Prolonged feeding duration may be caused by longer pre-attachment times [12].

Several attempts have been made to reduce the pre-attachment duration by increasing the attractiveness of the artificial membrane, for instance by the addition of animal hair extract, tick feces or tick feces extract, animal hair or rubbing the membrane on live animals [27,46,47,48]. For our study, we decided to use only cow hair extract, as other additions might increase contamination risk [49]. In addition, the CO_2_ level was set at 4% to stimulate tick activity [50]. Increased CO_2_ levels were previously reported to increase engorgement proportions and/or detachment weights of in vitro fed *D. reticulatus* [35] and *Amblyomma* ticks [22,27]. 

While the attachment proportions of nymphs in vitro in individual feeding units is relatively stable and fluctuated between 45–90%, stronger fluctuations were found for adults (0–100%) and larvae (12–95%). In this study, we observed a higher maximum attachment proportion for F_1_ adult ticks fed in a water bath (90 or 95%) compared to incubator-fed F_0_ or F_1_ adults, with attachment proportions of 20% and 75%, respectively. This might be explained by the presence of a natural light and circadian rhythm in the uncovered water bath. Another option could be an overstimulation by increased CO_2_ levels, which was also observed in previous adult in vitro feeding, albeit at a higher CO_2_ level of 10% [22]. However, as F_0_ ticks showed a lower attachment proportion than F_1_ ticks in both incubator and water bath, seasonality might also play a role, as F_0_ ticks were fed during winter and F_1_ ticks during summer. Another possible explanation might be an adaptation of ticks to in vitro feeding, as was for instance reported for mosquitos [51]. 

The complex feeding behavior and the long feeding duration of hard ticks make the evaluation of the effect of different artificial feeding conditions time-consuming, but it would be interesting to conduct further studies on the effect of factors, such as seasonality, tick age, and environmental conditions, have on the in vitro feeding success. 

### 4.2. Tick Weights

In our study, significantly lower detachment weights were observed for all in vitro fed life stages compared to in vivo fed ticks, although the mean engorgement weights of both in vitro (0.43 mg) and in vivo (0.53 mg) fed larvae were still in the range of previous reports of larvae fed on animals (0.373–0.563 mg) [52,53,54]. 

The lower engorgement weights of in vitro fed *I. ricinus* nymphs were also in agreement with findings from previous studies (means 2.8–3 mg) [19,40]. The in vitro data showed a bimodal distribution, which is common for *Ixodes* nymphs and is related to the sex of the adult, with nymphs that molt into females having a higher engorgement weight than nymphs that molt into males [55,56,57]. Differences between the sex ratios of adults (67 females: 90 males in vitro, 232 females: 197 males in vivo), could at least partially explain the lower mean engorgement weights of nymphs. 

The unfed F_1_ adult weights were significantly lower in in vitro experiments than in in vivo experiments (unfed: ♀ *p* < 0.0001, df = 97.74 ♂ *p* < 0.0001, df = 198.54). The lower weight of unfed adults may be caused by the accumulative effect of the reduced engorgement weights observed for larvae and nymphs, which may eventually have resulted in smaller adult ticks. The mean weight of unfed larvae (0.0223 mg) used to calculate the approximate number of larvae used for the in vivo feedings was also lower compared to a previous report (0.034 mg) [55]. To avoid manipulation of larvae for in vivo feeding, we used this measuring method to calculate the number of deployed larvae. In contrast, in vitro deployed ticks were able to count since a smaller number of larvae were used per unit compared to a calf’s ear.

Furthermore, feeding in a water bath vs. incubator environment appeared to have an impact on detachment weights of adult ticks. In the incubator, detachment weights of the F_1_ adults were on average 55 mg lower than the F_0_ adults, but in the water bath, F_1_ ticks showed similar detachment weights as F_0_ ticks (F_1_: 112 ± 38 mg vs. F_0_: 136 ± 45 mg). The availability of a natural light source and light-dark rhythm in the water bath might have been responsible for these differences. A larger sample size would be necessary to obtain a better understanding of the effects of different feeding environments on *I. ricinus* engorgement. For ticks fed on cattle, our findings correspond with previous findings concerning detachment weights [19,58]. 

### 4.3. Completion of Life Stages 

In our study, a larger proportion of in vitro fed larvae successfully fed and molted into nymphs (44%) than in vivo fed larvae (24%). Since bovine blood was used in the ATFS, we opted for cattle for all in vivo feedings even though larvae are commonly lab-reared on mice or rabbits [31,59]. As cattle may not be the preferred hosts of *I. ricinus* larvae, it is plausible that the feeding of larvae on cattle ears reduced their in vivo feeding success. 

In contrast, the proportion of nymphs that engorged in vitro (49%) was lower than in vivo (74%). This corroborates findings from previous experiments involving silicone membranes [19,40]. Experiments using animal skin membranes tend to produce higher engorgement proportions [41], presumably because ticks are more attracted to animal skin than to artificial silicone-based membranes. In our study, 75% of in vitro engorged nymphs molted successfully, which was similar to the molting success of in vivo engorged nymphs (72%) and higher than the in vitro *I. ricinus* molting success described in other studies [19,40]. However, the total outcome as proportion of molted adults from all fed nymphs was significantly lower in vitro (37%) compared to in vivo (54%). 

Similar deficits were observed for in vitro F_0_ adult feeding: the F_0_ generation of in vitro fed adult ticks showed significantly lower detachment, oviposition and outcome of viable larvae batches than the in vivo F_0_ generation. It appeared that this could be improved by feeding F_1_ adults in a water bath with a blood meal supplemented with vitamin B. Here no significant differences in detachment proportion and outcome of larvae producing females compared to in vivo fed females were observed. 

### 4.4. Strength and Limitations

The miniature 12-well plate ATFS version presents a material-saving opportunity, as it results in a reduction of the amount of blood required. This can be of particular interest to studies in which valuable compounds such as novel drug compounds or antibodies raised against tick antigens within studies focusing on the development of anti-tick vaccines are added to the blood meal to examine their effect on tick feeding [18,60,61,62]. An additional advantage of this ATFS is that it also facilitates the feeding of juvenile life stages, which cannot easily be fed using capillary- or tube-based feeding systems [63]. 

A major limitation of the ATFS is the long feeding durations and associated risk of contamination. To limit this risk, calves were used as blood donors instead of blood collected during exsanguination at an abattoir [35,49]. Furthermore, only sterile or autoclaved materials and blood meal ingredients were used. In general, contamination started appearing after ~7 days of in vitro feeding and manifested itself by darkening and foul smell of the blood meal, and an increased white-yellow mucus on the blood side of the membrane. Fungal contamination inside the feeding unit was enhanced by small leakage of the membrane that introduced blood into the unit. When recognized quickly, ticks could be manually detached, washed and introduced in a new feeding unit or the dried blood can be discarded from the unit. Nymphs in particular tended to cluster and attach in corners of the feeding units, which may increase the risk of leakage (Supplementary Materials Figure S1, Video S1). 

To further prevent bacterial growth, gentamicin was added to the blood meal, but this could not completely prevent contamination. Previous research suggests that antibiotics have a negative influence on the tick microbiome and consequently on tick fitness and fecundity [27,30,35]. Studies showed that after antibiotic treatment of ticks, hosts or blood meals, the endosymbiont density and/or composition changed, which resulted in a reduction of reproduction fitness [29,30,61,64]. or a decrease in development [65]. The use of antibiotics in the blood meal in this study could therefore explain the relatively low weights and proportions observed in the in vitro experiments. The negative impact of antibiotics on tick endosymbionts that may result in reduced tick fitness can be explained by the role that these endosymbionts appear to play in vitamin B pathways of obligate hematophagous parasites. It was previously shown that decreasing levels of tick endosymbionts, for instance as a result of antibiotic treatment, had an impact on vitamin B synthesis and tick survival [29,30,66,67,68]. Tick endosymbionts such as *Coxiella*-like, *Rickettsia*-like, but also *Wolbachia, Midichloria*, or *Francisella* are in focus of the vitamin B pathways [29,69,70]. To prevent a lack of vitamin B available to the tick caused by a disruption of the tick’s endosymbionts, a vitamin B supplementation to the ticks’ diet has been suggested [29]. In our study, one group of F_1_ adults therefore received a vitamin B supplement as previously described [29,71]. The vitamin B supplement group continually showed higher detachment weights, egg masses, and higher detachment and oviposition proportions than the non-supplemented group, improving the in vitro F_0_ adult feeding and rendering it nearly as successful as the in vivo group. Fewer attached females died during feeding in the vitamin B supplemented group (0/20) than in the non-supplemented group (9/20). Dead females were observed with black and sometimes spherical bodies similar to previous descriptions [67]. Furthermore, for F_1_ adults, we observed a positive effect on weights and proportions. In contrast, this clear effect on weights was not seen for F_0_ adults (data not shown). Nevertheless, we observed a less number of dead ticks at the end of F_0_ adult feeding experiments with vitamin B supplementation and a positive effect on the proportion on females producing viable larvae batches (data not shown). It should be noted that the addition of antibiotics to the blood meal may also impact the growth of pathogens, which should be considered in studies aiming to study tick-pathogen interactions. 

While the positive effect of vitamin B supplementation in an ATFS without antibiotics may be negligible [29], further research regarding its effect on the feeding of larvae and nymphs would be justified. 

## 5. Conclusions

All consecutive life stages of the hard tick *I. ricinus* were fed by artificial feeding and compared to data collected from experimental *I. ricinus* infestations on cattle. The data showed that the in vitro feeding of F_1_ larvae was as effective as larvae feed in vivo on cattle, but the in vitro feeding of F_1_ nymphs and adults was not as successful as the in vivo feeding on cattle. The complex feeding behavior of ixodid ticks such as *I. ricinus* and the prolonged duration of in vitro feeding resulted in an increased risk of contamination in the ATFS over time. The use of sterile blood, a sterile workflow and the supplementation of antibiotics to the blood meal may delay contamination, but the effect of antibiotics on tick fecundity requires further evaluation. The addition of vitamin B components to the diet of F_1_ adults appeared to have a positive effect on tick feeding and fecundity. This suggests that vitamin B supplementation is essential for *I. ricinus* ticks previously exposed to antibiotic treatment, probably due to the detrimental effect of antibiotics on nutritive tick symbionts that would otherwise provide ticks with these vitamins. 

## Figures and Tables

**Table 1:** Artificial feeding of F_0_ and F_1_ females in comparison to in vivo. Artificial feeding of 30 F_0_-female *I. ricinus* ticks from in vivo origin compared to artificial feeding of F_1_-in vitro generation female ticks in water bath with and without vitamin B components and to in vivo tick feeding on calves.

	Adult Feeding Experiment				Statistical Analyses (Test, *p*-Value, df = Degrees of Freedom)		
	In Vitro			In Vivo *n* = 450			
	F_0_ *n* = 30	F_1_			In Vitro F_0_ to In Vivo	In Vitro F_1_ (Vitamin B) to In Vivo	In Vitro F_0_ to In Vitro F_1_ (Vitamin B)
		No Vitamin B, *n* = 10	Vitamin B, *n* = 21				
Maximum attachment (%)	20 (CI: 9–37)	90 (CI: 59–98)	95 (CI: 77–99)	---	---	---	Z-test, *p* < 0.0001, df = 1, χ^2^ = 35,119
Detachment (%)	20 (CI: 9–37)	50 (CI: 23–76)	90 (CI: 71–97)	71 (CI: 66–75)	Z-test, *p* < 0.0001, df = 1, χ^2^ = 33.312	Z-test, *p* = 0.0513, df = 1, χ^2^ = 3.8	Z-test, *p* < 0.0001, df = 1, χ^2^ = 24.55
Mean duration until detachment (days)	11.5 ± 0.8 (CV: 7.3)	11 ± 0.7 (CV: 6.4)	12.3 ± 2.5 (CV: 20.1)	6.9 ± 1.5 (CV: 21.3)	MWU, *p* < 0.0001, W = 1860	MWU, *p* < 0.0001, W = 5893.5	MWU, *p* = 0.921, W = 59
Mean detachment weight (mg)	136 ± 44.9 (CI: 89–183.3)	112 ± 37.5 (CI: 72.5–151)	180 ± 64.1 (CI: 149–211)	231 ± 72.3 (CI: 222.9–238.9)	*t*-test, *p* = 0.003, df = 5.5	*t*-test *p* = 0.003, df = 20.832	*t*-test, *p* = 0.088, df = 12.109
Oviposition of all detached ticks (%)	67 (CI: 29–90)	40 (CI: 11–77)	79 (CI: 56–91)	91 (CI: 87–94)	Z-test, *p* = 0.0452, df = 1, χ^2^ = 4.011	Z-test, *p* = 0.088, df = 1, χ^2^ = 2.912	Z-test, *p* = 0.539, df = 1, χ^2^ = 0.377
Mean duration of oviposition (days)	22.8 ± 11.4 (CV: 49.9)	19 ± 4.2 (CV: 22.3)	12.8 ± 5.4 (CV: 36.7)	35.8 ± 16.2 (CV: 45.1)	MWU, *p* = 0.128, W = 322.5	MWU, *p* < 0.0001, W = 407	MWU, *p* = 0.071, W = 51.5
Mean egg mass (mg)	46 ± 27.8 (CI: 1.7–90.2)	7.13 ± 3.37 (CI: 0–37.4)	56 ± 27.2 (CI: 40.5–71.9)	116 ± 39.5 (CI: 111–121)	*t*-test, *p* = 0.0137, df = −3.195	*t*-test, *p* < 0.0001, df = 16.215	*t*-test, *p* = 0.544, df = 4.781
Mean egg conversion factor	28.5 ± 10.5 (CI: 11.8–45.2)	6.5 ± 3.5 (CI: 0–38.3)	30.4 ± 12.3 (CI: 23.3–37.4)	47.5 ± 10.2 (CI: 46.2–48.7)	*t*-test, *p* = 0.0351, df = 3.0917	*t*-test *p* = 0.0002, df = 14.022	*t*-test, *p* = 0.775, df = 5.621
Larvae producing females per egg batches (%)	75 (CI: 30–95)	100 (CI: 34–100)	80 (CI: 54–93)	81 (CI: 76–85)	Z-test, *p* = 0.745, df = 1, χ^2^ = 0.1056	Z-test, *p* = 0.894, df = 1, χ^2^ = 0.018	Z-test, *p* = 0.828, df = 1, χ^2^ = 0.048
Mean larvae hatch duration (days)	59.7 ± 1.5 (CV: 2.6)	69.5 ± 0.7 (CV: 1)	64.4 ± 12.8 (CV: 19.9)	67.2 ± 6.4 (CV: 9.5)	MWU, *p* = 0.071, W = 115	MWU, *p* = 0.011, W = 661	MWU, *p* = 0.995, W = 16.5
Mean larvae hatching step (1–5)	3 (IQR: 0)	3 (IQR: 2)	5 (IQR: 2)	5 (IQR: 2)	MWU, *p* = 0.072, W = 165	MWU, *p* = 0.625, W = 960.5	MWU, *p* = 0.282, W = 7.5
Larvae prod. females per all fed females (%)	10 (CI: 3–26)	20 (CI: 5–51)	57 (CI: 36–76)	52 (CI: 47–57)	Z-test, *p* < 0.0001, df = 1, χ^2^ = 20.268	Z-test, *p* = 0.673, df = 1, χ^2^ = 0.177	Z-test, *p* = 0.0003, df = 1, χ^2^ = 13.224

Data on individual ticks, mean values ± standard deviation and 95% confidence interval (CI) or coefficient of variation (CV). For larvae hatch steps, median and interquartile range (IQR) was computed. Statistical tests were performed by Z-test for proportions and by either *t*-test with Welch correction for normal distribution or by Mann–Whitney U (MWU) test for non-normal distribution, respectively. Additionally, degrees of freedom (df) and chi-square (χ^2^) were computed.

**Table 2:** Artificial feeding of *I. ricinus* larvae.

	Larvae Feeding Experiment		Statistical Analyses (Test, *p*-Value, df = Degrees of Freedom) In Vitro to In Vivo
	In Vitro *n* = 1003	In Vivo *n* = 11,737	
Attachment on day 3 (%)	60 (CI: 57–63)	---	---
Engorgement (%)	55 (CI: 52–58)	41 (CI: 40–42)	Z-test, *p* < 0.0001, df = 1, χ^2^ = 76.44
Mean duration until first engorged tick (days) *	4.8 ± 0.6 (CV: 12.5)	3 (CV: 0)	MWU, *p* = 0.001, W = 55
Mean duration of feeding experiment (days) *	9.6 ± 1.3 (CV: 13.5)	3.8 ± 0.4 (CV: 11.8)	MWU, *p* = 0.0018, W = 55
Mean engorgement (mg) **	0.43 ± 0.02 (CV: 4.8)	0.53 ± 0.03 (CV: 6.4)	MWU, *p* = 0.0003, W = 1
Molting per engorged tick (%)	83 (CI: 76–84)	59 (CI: 57–60)	Z-test, *p* < 0.0001, df = 1, χ^2^ = 97
Proportion of deployed larvae reaching the next life stage (%)	44 (CI: 41–48)	24 (CI: 23–25)	Z-test, *p* < 0.0001, df = 1, χ^2^ = 199.15

*n* = number of used ticks, mean values ± standard deviation and a 95% confidence interval (CI) or coefficient of variation (CV). Statistical tests were performed by Z-test for proportions and by either *t*-test with Welch correction for normal distribution or by Mann–Whitney U (MWU) test for non-normal distribution, respectively. Additionally, degrees of freedom (df) and chi-square (χ^2^) were computed. * = per feeding unit in vitro (*n* = 12) or per experiment in vivo (*n* = 5); ** = per weighted larvae batch (in vitro *n* = 8, in vivo *n* = 12).

**Table 3:** Artificial feeding of *I. ricinus* nymphs.

	Nymph Feeding Experiment		Statistical Analyses (Test, *p*-Value, df = Degrees of Freedom) In Vitro to In Vivo
	In Vitro *n* = 426	In Vivo *n* = 800	
Maximum attachment (%)	68 (CI: 63–73)	---	---
Engorgement (%)	49 (CI: 44–54)	74 (CI: 70–86)	Z-test, *p* < 0.0001, df = 1, χ^2^ = 71.67
Mean duration until first engorged tick (days) *	6.9 ± 1.4 (CV: 20.7)	2.9 ± 0.4 (CV: 2.5)	MWU, *p* < 0.0001, W = 119
Mean duration of feeding experiment (days) *	11.4 ± 2.9 (CV: 25)	5.3 ± 0.8 (CV: 14.3)	MWU, *p* < 0.0001, W = 171
Mean engorgement weight (mg)	2.82 ± 0.84 (CV: 29.7)	3.32 ± 0.96 (CV: 28.9)	MWU, *p* < 0.0001, W = 41,300
Molting rate per engorged tick (%)	75 (CI: 68–80)	73 (CI: 69–76)	Z-test, *p* = 0.6117, df = 1, χ^2^ = 0.26
Rate of females per molted adults (%)	43 (CI: 35–51) ♀ = 67; ♂ = 90	54 (CI: 49–59) ♀ = 232; ♂ = 197	Z-test, *p* = 0.0145, df = 1, χ^2^ = 5.98
Mean weight of female& male (mg)	♀ 1.32 ± 0.3 (CI: 1.25–1.39) ♂ 0.81 ± 0.16 (CI: 0.77–0.84)	♀ 1.68 ± 0.25 (CI: 1.65–1.72) ♂ 0.98 ± 0.18 (CI: 0.95–1)	♀: *t*-test, *p* < 0.0001, df = 97.74 ♂: *t*-test, *p* < 0.0001, df = 198.54
Proportion of deployed nymphs reaching the next life stage (%)	37 (CI: 32–42)	54 (CI: 50–57)	Z-test, *p* < 0.0001, df = 1, χ^2^ = 31.33

*n* = number of used ticks, mean values ± standard deviation and a 95% confidence interval (CI) or coefficient of variation (CV). Statistical tests were performed by Z-test for proportions and by either *t*-test with Welch correction for normal distribution or by Mann–Whitney U (MWU) test for non-normal distribution, respectively. Additionally, degrees of freedom (df) and chi-square (χ^2^) were computed. * = per feeding unit in vitro (*n* = 22) or per experiment in vivo (*n* = 7).

**Table 4:** Detachment weights and egg masses of in vitro and in vivo fed adult ticks analyzed by four linear mixed-effect models (LMMs) using in vitro F_1_-adults + vitamin B + water bath as reference group.

	Group	Estimates (mg)	95% CI	*n*	*p*
**Detachment weight**	F_1_ + vitamin B + water bath	reference			
	F_0_, no vitamin B + incubator	−43.52	(−95.64, 8.61)	31	0.102
	F_1_, no vitamin B + incubator	−124.68	(−238.88, −0.48)	31	0.032
	F_1_, no vitamin B + water bath	−56.48	(−112.43, −0.54)	31	0.048
	in vivo	+50.51	(4.55, 96.48)	338	0.031
**Egg mass**	F_0_, no vitamin B + incubator	−10.22	(−39.65, 19.2)	21	0.496
	F_1_, no vitamin B + incubator	−42	(−95.73, 11.72)	21	0.121
	F_1_, no vitamin B + water bath	−49.09	(−88.33, −9.86)	21	0.014
	in vivo	+59.5	(30.04, 88.96)	266	<0.001

## Data Availability

The data presented in this study are available upon request from the corresponding author.

## Supplementary Materials

The following are available online at https://www.mdpi.com/article/10.3390/vaccines9040385/s1, Figure S1: Impressions on artificial feedings, Video S1: Artificial feeding of *I. ricinus* nymphs.

## References

[1] Bedford G. (1931). *Nuttalliella namaqua*, a new genus and species of tick. Parasitology.

[2] Mans B.J., De Klerk D., Pienaar R., Latif A.A. (2011). *Nuttalliella namaqua*: A living fossil and closest relative to the ancestral tick lineage: Implications for the evolution of blood-feeding in ticks. PLoS ONE.

[3] Guglielmone A.A., Robbins R.G., Apanaskevich D.A., Petney T.N., Estrada-Pena A., Horak I.G., Shao R., Barker S.C. (2010). The Argasidae, Ixodidae and Nuttalliellidae (Acari: Ixodida) of the world: A list of valid species names. Zootaxa.

[4] Jongejan F., Uilenberg G. (2004). The global importance of ticks. Parasitology.

[5] Mac S., da Silva S.R., Sander B. (2019). The economic burden of Lyme disease and the cost-effectiveness of Lyme disease interventions: A scoping review. PLoS ONE.

[6] Petney T.N., Pfaeffle M.P., Skuballa J.D. (2012). An annotated checklist of the ticks (Acari: Ixodida) of Germany. Syst. Appl. Acarol..

[7] Brugger K., Boehnke D., Petney T., Dobler G., Pfeffer M., Silaghi C., Schaub G.A., Pinior B., Dautel H., Kahl O. (2016). A Density Map of the Tick-Borne Encephalitis and Lyme Borreliosis Vector Ixodes ricinus (Acari: Ixodidae) for Germany. J. Med Entomol..

[8] Milne A. (1949). The ecology of the sheep tick, Ixodes ricinus L. Host relationships of the tick: Part 1. Review of previous work in Britain. Parasitology.

[9] Estrada-Peña A., Jongejan F. (1999). Ticks feeding on humans: A review of records on human-biting Ixodoidea with special reference to pathogen transmission. Exp. Appl. Acarol..

[10] Hokama Y., Lane R.S., Howarth J.A. (1987). Maintenance of adult and nymphal Ornithodoros coriaceus (Acari: Argasidae) by artificial feeding through a parafilm membrane. J. Med Entomol..

[11] Schwan E.V., Hutton D., Shields K.J., Townson S. (1991). Artificial feeding and successful reproduction in Ornithodoros moubata moubata (Murray, 1877) (Acarina: Argasidae). Exp. Appl. Acarol..

[12] Nijhof A.M., Tyson K.R. (2018). In vitro Feeding Methods for Hematophagous Arthropods and Their Application in Drug Discovery. Ectoparasites.

[13] Romano D., Stefanini C., Canale A., Benelli G. (2018). Artificial blood feeders for mosquito and ticks-Where from, where to?. Acta Trop..

[14] Rutledge L.C., Ward R.A., Gould D.J. (1964). Studies on the Feeding Response of Mosquitos to Nutritive Solutions in a New Membrane Feeder. Mosq. News.

[15] Campbell E.M., Burdin M., Hoppler S., Bowman A.S. (2010). Role of an aquaporin in the sheep tick *Ixodes ricinus*: Assessment as a potential control target. Int. J. Parasitol..

[16] Abraham N.M., Liu L., Jutras B.L., Yadav A.K., Narasimhan S., Gopalakrishnan V., Ansari J.M., Jefferson K.K., Cava F., Jacobs-Wagner C. (2017). Pathogen-mediated manipulation of arthropod microbiota to promote infection. Proc. Natl. Acad. Sci. USA.

[17] Trentelman J.J., Kleuskens J.A., van de Crommert J., Schetters T.P. (2017). A new method for in vitro feeding of *Rhipicephalus australis* (formerly *Rhipicephalus microplus*) larvae: A valuable tool for tick vaccine development. Parasites Vectors.

[18] Contreras M., Alberdi P., Fernandez De Mera I.G., Krull C., Nijhof A., Villar M., De La Fuente J. (2017). Vaccinomics Approach to the Identification of Candidate Protective Antigens for the Control of Tick Vector Infestations and *Anaplasma phagocytophilum* Infection. Front. Cell. Infect. Microbiol..

[19] Knorr S., Anguita J., Cortazar J.T., Hajdusek O., Kopacek P., Trentelman J.J., Kershaw O., Hovius J.W., Nijhof A.M. (2018). Preliminary Evaluation of Tick Protein Extracts and Recombinant Ferritin 2 as Anti-tick Vaccines Targeting *Ixodes ricinus* in Cattle. Front. Physiol..

[20] Antunes S., Merino O., Mosqueda J., Moreno-Cid J.A., Bell-Sakyi L., Fragkoudis R., Weisheit S., de la Lastra J.M.P., Alberdi P., Domingos A. (2014). Tick capillary feeding for the study of proteins involved in tick-pathogen interactions as potential antigens for the control of tick infestation and pathogen infection. Parasites Vectors.

[21] Totze R. (1933). Beiträge zur Sinnesphysiologie der Zecken. Z. Für Vgl. Physiol..

[22] Voigt W.P., Young A.S., Mwaura S.N., Nyaga S.G., Njihia G.M., Mwakima F.N., Morzaria S.P. (1993). In vitro feeding of instars of the ixodid tick *Amblyomma variegatum* on skin membranes and its application to the transmission of *Theileria mutans* and *Cowdria ruminatium*. Parasitology.

[23] DeVries Z.C., Mick R., Schal C. (2016). Feel the heat: Activation, orientation and feeding responses of bed bugs to targets at different temperatures. J. Exp. Biol..

[24] Lees A.D. (1948). The Sensory Physiology of the Sheep Tick, *Ixodes ricinus* L. J. Exp. Biol..

[25] Matuschka F.-R., Richter D., Fischer P., Spielman A. (1990). Time of repletion of subadult *Ixodes ricinus* ticks feeding on diverse hosts. Parasitol. Res..

[26] Waladde S., Rice M., Obenchain F.D., Galun R. (1982). The sensory basis of tick feeding behaviour. Physiology of Ticks.

[27] Kuhnert F., Diehl P.A., Guerin P.M. (1995). The life-cycle of the bont tick *Amblyomma hebraeum* in vitro. Int. J. Parasitol..

[28] Bohme B., Krull C., Clausen P.H., Nijhof A.M. (2018). Evaluation of a semi-automated in vitro feeding system for *Dermacentor reticulatus* and *Ixodes ricinus* adults. Parasitol. Res..

[29] Duron O., Morel O., Noel V., Buysse M., Binetruy F., Lancelot R., Loire E., Menard C., Bouchez O., Vavre F. (2018). Tick-Bacteria Mutualism Depends on B Vitamin Synthesis Pathways. Curr. Biol..

[30] Zhong J., Jasinskas A., Barbour A.G. (2007). Antibiotic treatment of the tick vector *Amblyomma americanum* reduced reproductive fitness. PLoS ONE.

[31] Levin M.L., Schumacher L.B. (2016). Manual for maintenance of multi-host ixodid ticks in the laboratory. Exp Appl Acarol.

[32] Krober T., Guerin P.M. (2007). An in vitro feeding assay to test acaricides for control of hard ticks. Pest Manag. Sci. Former. Pestic. Sci..

[33] Krober T., Guerin P.M. (2007). In vitro feeding assays for hard ticks. Trends Parasitol..

[34] de Carvalho Ferreira H.C., Tudela Zuquete S., Wijnveld M., Weesendorp E., Jongejan F., Stegeman A., Loeffen W.L. (2014). No evidence of African swine fever virus replication in hard ticks. Ticks Tick-Borne Dis..

[35] Krull C., Bohme B., Clausen P.H., Nijhof A.M. (2017). Optimization of an artificial tick feeding assay for *Dermacentor reticulatus*. Parasites Vectors.

[36] Winkham H. (2016). ggplot2: Elegant Graphics for Data Analysis.

[37] Fourie J.J., Evans A., Labuschagne M., Crafford D., Madder M., Pollmeier M., Schunack B. (2019). Transmission of *Anaplasma phagocytophilum* (Foggie, 1949) by *Ixodes ricinus* (Linnaeus, 1758) ticks feeding on dogs and artificial membranes. Parasites Vectors.

[38] Andrade J.J., Xu G., Rich S.M. (2014). A silicone membrane for in vitro feeding of *Ixodes scapularis* (Ixodida: Ixodidae). J. Med Entomol..

[39] Oliver J.D., Lynn G.E., Burkhardt N.Y., Price L.D., Nelson C.M., Kurtti T.J., Munderloh U.G. (2016). Infection of Immature *Ixodes scapularis* (Acari: Ixodidae) by Membrane Feeding. J. Med Entomol..

[40] Körner S., Makert G.R., Mertens-Scholz K., Henning K., Pfeffer M., Starke A., Nijhof A.M., Ulbert S. (2020). Uptake and fecal excretion of *Coxiella burnetii* by *Ixodes ricinus* and *Dermacentor marginatus* ticks. Parasites Vectors.

[41] Bonnet S., Jouglin M., Malandrin L., Becker C., Agoulon A., L’Hostis M., Chauvin A. (2007). Transstadial and transovarial persistence of *Babesia divergens* DNA in *Ixodes ricinus* ticks fed on infected blood in a new skin-feeding technique. Parasitology.

[42] Gray J. (1991). The development and seasonal activity of the tick *Ixodes ricinus*: A vector of Lyme borreliosis. Rev. Med Vet. Entomol..

[43] Kocan K.M., de la Fuente J., Coburn L.A. (2015). Insights into the development of *Ixodes scapularis*: A resource for research on a medically important tick species. Parasites Vectors.

[44] Musyoki J.M., Osir E.O., Kiara H.K., Kokwaro E.D. (2004). Comparative studies on the infectivity of *Theileria parva* in ticks fed in vitro and those fed on cattle. Exp. Appl. Acarol..

[45] González J., Valcárcel F., Aguilar A., Olmeda A. (2017). In vitro feeding of *Hyalomma lusitanicum* ticks on artificial membranes. Exp. Appl. Acarol..

[46] Waladde S.M., Ochieng S.A., Gichuhi P.M. (1991). Artificial-membrane feeding of the ixodid tick, *Rhipicephalus appendiculatus*, to repletion. Exp. Appl. Acarol..

[47] Habedank B., Hiepe T. (1993). In-vitro feeding of ticks, *Dermacentor nuttalli;* Olenev 1928 (Acari: Ixodidae) on a silicon membrane. Dermatol. Mon..

[48] Grenacher S., Krober T., Guerin P.M., Vlimant M. (2001). Behavioural and chemoreceptor cell responses of the tick, *Ixodes ricinus*, to its own faeces and faecal constituents. Exp. Appl. Acarol..

[49] Kuhnert F. (1996). Feeding of Hard Ticks In Vitro: New Perspectives for Rearing and for the Identification of Systemic Acaricides. ALTEX.

[50] van Duijvendijk G., Gort G., Sprong H., Takken W. (2017). Behavioural responses of *Ixodes ricinus* nymphs to carbon dioxide and rodent odour. Med Vet. Entomol..

[51] Ross P.A., Lau M.-J., Hoffmann A.A. (2019). Does membrane feeding compromise the quality of *Aedes aegypti* mosquitoes?. PLoS ONE.

[52] Matuschka F.-R., Spielman A. (1992). Loss of Lyme disease spirochetes from *Ixodes ricinus* ticks feeding on European blackbirds. Exp. Parasitol..

[53] Talleklint L., Jaenson T.G.T. (1997). Infestation of mammals by *Ixodes ricinus* ticks (Acari: Ixodidae) in south-central Sweden. Exp. Appl. Acarol..

[54] Hughes V.L., Randolph S.E. (2001). Testosterone depresses innate and acquired resistance to ticks in natural rodent hosts: A force for aggregated distributions of parasites. J. Parasitol..

[55] Balashov Y.S. (1972). Bloodsucking ticks (Ixodoidea)-vectors of disease in man and animals. Misc. Publ. Entomol. Soc. Am..

[56] Kahl O., Hoff R., Knulle W. (1990). Gross morphological changes in the salivary glands of *Ixodes ricinus* (Acari, Ixodidae) between bloodmeals in relation to active uptake of atmospheric water vapour. Exp. Appl. Acarol..

[57] Hu R., Rowley W.A. (2000). Relationship between weights of the engorged nymphal stage and resultant sexes in *Ixodes scapularis* and *Dermacentor variabilis* (Acari: Ixodidae) ticks. J. Med Entomol..

[58] Taylor S., Kenny J. (1990). An ivermectin sustained release bolus in cattle: Its effects on the tick *Ixodes ricinus*. Med Vet. Entomol..

[59] Jaenson T.G., Talleklint L., Lundqvist L., Olsen B., Chirico J., Mejlon H. (1994). Geographical distribution, host associations, and vector roles of ticks (Acari: Ixodidae, Argasidae) in Sweden. J. Med Entomol..

[60] Trentelman J.J.A., Teunissen H., Kleuskens J., van de Crommert J., de la Fuente J., Hovius J.W.R., Schetters T.P.M. (2019). A combination of antibodies against Bm86 and Subolesin inhibits engorgement of *Rhipicephalus australis* (formerly *Rhipicephalus microplus*) larvae in vitro. Parasites Vectors.

[61] Lu S., Parizi L.F., Torquato R.J.S., Vaz Junior I.S., Tanaka A.S. (2019). Novel pseudo-aspartic peptidase from the midgut of the tick *Rhipicephalus microplus*. Sci. Rep..

[62] Kemp D.H., Agbede R.I., Johnston L.A., Gough J.M. (1986). Immunization of cattle against *Boophilus microplus* using extracts derived from adult female ticks: Feeding and survival of the parasite on vaccinated cattle. Int. J. Parasitol..

[63] Lew-Tabor A.E., Bruyeres A.G., Zhang B., Valle M.R. (2014). *Rhipicephalus (Boophilus) microplus* tick in vitro feeding methods for functional (dsRNA) and vaccine candidate (antibody) screening. Ticks Tick-Borne Dis..

[64] Zhang C.-M., Li N.-X., Zhang T.-T., Qiu Z.-X., Li Y., Li L.-W., Liu J.-Z. (2017). Endosymbiont CLS-HI plays a role in reproduction and development of *Haemaphysalis longicornis*. Exp. Appl. Acarol..

[65] Guizzo M.G., Parizi L.F., Nunes R.D., Schama R., Albano R.M., Tirloni L., Oldiges D.P., Vieira R.P., Oliveira W.H.C., Leite M.D.S. (2017). A *Coxiella* mutualist symbiont is essential to the development of *Rhipicephalus microplus*. Sci. Rep..

[66] Smith T.A., Driscoll T., Gillespie J.J., Raghavan R. (2015). A Coxiella-like endosymbiont is a potential vitamin source for the Lone Star tick. Genome Biol. Evol..

[67] Duron O., Gottlieb Y. (2020). Convergence of nutritional symbioses in obligate blood feeders. Trends Parasitol..

[68] Hosokawa T., Koga R., Kikuchi Y., Meng X.Y., Fukatsu T. (2010). *Wolbachia* as a bacteriocyte-associated nutritional mutualist. Proc. Natl. Acad. Sci. USA.

[69] Olivieri E., Epis S., Castelli M., Boccazzi I.V., Romeo C., Desirò A., Bazzocchi C., Bandi C., Sassera D. (2019). Tissue tropism and metabolic pathways of *Midichloria mitochondrii* suggest tissue-specific functions in the symbiosis with *Ixodes ricinus*. Ticks Tick-Borne Dis..

[70] Nikoh N., Hosokawa T., Moriyama M., Oshima K., Hattori M., Fukatsu T. (2014). Evolutionary origin of insect–*Wolbachia* nutritional mutualism. Proc. Natl. Acad. Sci. USA.

[71] Lake P., Friend W.G. (1968). The use of artificial diets to determine some of the effects of *Nocardia rhodnii* on the development of *Rhodnius prolixus*. J. Insect Physiol..

